# Antenatal care service quality increases the odds of utilizing institutional delivery in Bahir Dar city administration, North Western Ethiopia: A prospective follow up study

**DOI:** 10.1371/journal.pone.0192428

**Published:** 2018-02-08

**Authors:** Tadese Ejigu Tafere, Mesganaw Fanthahun Afework, Alemayehu Worku Yalew

**Affiliations:** 1 School of Public Health (SPH), College of Health Sciences, Addis Ababa University, Addis Ababa, Ethiopia; 2 School of Public Health, College of Medicine and Health Sciences, Bahir Dar University, Bahir Dar, Ethiopia; 3 School of Public Health, College of Health Sciences, Addis Ababa University, Addis Ababa, Ethiopia; Dhaka University, BANGLADESH

## Abstract

**Background:**

In Ethiopia, more than 62% of pregnant women attend antenatal care at least once, yet only about one in four women give birth at health facility. This gap has fueled the need to investigate on the quality of ANC services at public health facilities and its link with the use of institutional delivery.

**Objective:**

To assess the linkage between ANC quality and the use of institutional delivery among pregnant women attending ANC at public health facilities of BDR City Administration

**Methods:**

A facility based prospective follow up study was conducted. and nine hundred seventy pregnant women with gestational age ≤ 16 weeks who came for their first ANC visit were enrolled.Women were followed from their first ANC visit until delivery. Longitudinal data was collected during consultation with ANC providers using structured observation checklist. ANC service was considered as acceptable quality if women received ≥75^th^ percentile of the essential ANC services. Generalized Estimating Equation (GEE) was carried out to control cluster effect among women who received ANC in the same facility.

**Results:**

Among 823 pregnant women who completed follow up, only about one third (27.6%) received acceptable quality of ANC services. In one health facility syphilis test was not done at all for the last two years. The odds of giving birth at health institution among pregnant women who received acceptable ANC quality service was about 3.38 times higher than among pregnant women who received unacceptable ANC quality service (AOR = 3.38, 95% CI: 1.67, 6.83).

**Conclusion and recommendation:**

In this study the quality of ANC service provision in public health facilities was compromised/low. Provision of quality ANC service had a great role in promoting institutional delivery. Therefore the local authorities at each level of health sector or the nongovernmental organizations working to improve maternal health need to provide training on focused antenatal care protocol for ANC providers.

## Introduction

Care during pregnancy is important for the health of the mother and the development of the unborn baby. Antenatal care is a crucial time to promote healthy behaviors and parenting skills. World Health Organization (WHO) envisions “every pregnant woman and newborn to receive quality care throughout the pregnancy, childbirth and postnatal period. However, approximately 303 000 women died as a result of pregnancy and childbirth-related complications in 2015; 99% in low-resource settings, with Sub-Saharan Africa (SSA) countries alone accounting for roughly 66% and most can be prevented. The lifetime risk of maternal mortality is estimated at 1 in 36 in SSA, contrasting 1 in 4900 in developed countries. These showed the presence of greater disparities in maternal health care worldwide [1–4].

There is evidenced that effective interventions exist at reasonable cost for the prevention or treatment of virtually all life-threatening maternal and neonatal complications. These interventions include comprehensive reproductive health care; skilled care for all pregnant women, especially during delivery; and emergency obstetric care for all women and infants with life threatening complications. Yet, in spite of the global efforts to improve maternal health, the maternal mortality ratio (MMR) fell by approximately 44% over the past 25 years; this falls short of the MDG5 target reduction of at least 75% in MMR [5, 6].

Although the percentage of women attending Focused Antenatal Care (FANC) for at least once generally tends to be satisfactory even in low-income countries, maternal and neonatal mortality remain high. Similarly, in Ethiopia a relatively high ANC coverage coexists with low institutional delivery service coverage (about three-fourth the Ethiopian mothers are giving birth at home), high maternal mortality ratio (MMR) and neonatal mortality rate (NMR) which are 412/ 100,000 and 29/1000 live births respectively; that is 4 maternal deaths for every 1,000 live births [7, 8].

One of the four programmatic areas of the Ethiopia’s innovative Health Extension Program (HEP) which is staffed with two health extension workers (HEW) at each health post is to improve family health (ANC, delivery care, postnatal care and immunization services). The HEP contributes for the quality of ANC by regular mapping of households with pregnant women; referral linkage for ANC to primary health care and through provision of individual counseling on nutrition, breastfeeding, immunization, ITN use, family planning and importance of institutional delivery [9].

However, little conceptual or empirical work exists on the measurement of ANC quality and its role on promoting the use of institutional delivery service in Ethiopia and other low-income countries. Previous studies that try to assess ANC quality in the country were considering the frequency of ANC visit rather than the contents of the services and the information provided to pregnant women during their ANC visits. Therefore the aim of this study was to assess the link between antenatal care quality in terms of the contents of the ANC services and the use of institutional delivery.

## Methods

### Study design, setting and population

A facility based prospective follow up study was conducted from October 2015 to August 2016 in Bahir Dar City Administration, Amhara Regional State, which is located in the North West part of Ethiopia. According to the Amhara National Regional State Bureau of Finance and Economic Development report, the projected population by 2015/16 was 297,775 (80.5% urban Vs 19.5% rural), of these 156,515 (52.6%) were females & there were 3,300 eligible pregnant women.

The study was conducted on seven public health facilities, one hospital and six health centers. Currently two public hospitals and ten public health centers are providing maternal health care services but at the beginning of the data collection period of this study, one of the hospitals and four of the health centers were new and they were not included in the studied health facilities due to less flow of ANC service users.

All selected first visit pregnant women who were voluntary to participate; whose gestational age was ≤16weeks and on ANC follow up during data collection period were included in the study. Those who were seriously sick or referred from other facilities were excluded.

### Sample size and sampling technique

The sample size was calculated by Epi-info version 3.5. with the following assumptions: the proportion of women who gave birth at health facilities among those who received unacceptable antenatal care quality service was 16.5% from previous literature [10],95% confidence level, 80% power, 2:1 ratio of unexposed to exposed (those who received un acceptable ANC quality service to those who received acceptable quality of ANC) and 10% non response rate. Accordingly, the minimum sample size required for the study was 970 (647 non exposed & 323 exposed participants).

Proportional to size allocation was made to achieve the desired sample size of pregnant women from each selected public health facility based on the average number of ANC users in the most recent quarterly report of each health facilities (271 from Bahir Dar H/C, 161 from Felege-Hiwot referral Hospital, 154 from Han HC, 132 from Abay-mado HC, 114 from Shimbit HC, 94 from Meshenti and 42 from Zenzelma HC)

Study participants at each health facility were selected using systematic random sampling technique during the data collection period until the required sample size at each health facility was obtained. The sampling interval (k = 3) was calculated by dividing the source population to the total sample size (970) and this interval was used in all facilities to select study participants. The first client was selected by simple random sampling technique among the first three ANC service users.

### Data collection process

Pregnant women were followed from their first visit till they give birth.

A structured observation check list of 88 items developed based on FANC protocol was used to see the routine ANC practice for each visit. The components of ANC services assessed were categorized into three groups: (i) Comprehensive history taking (36 items) (ii); Measurements, tests and treatments (29 items) and (iii) Counseling/ provision of information (23 items).

For first visit:detailed history taking about past and current pregnancy, clinical examination laboratory testing, provision of information; distribution of drugs (TT and iron) and schedule of return visit were assessed.

For second visit: history of complaints in current pregnancy; assessment for fetal heart beat in addition to the clinical examination, information provision & drug distribution (TT and iron) and schedule of return visit were assessed.

For third visit: in addition to the procedures done in the second visit; updating hemoglobin test and prescribing mebenedazol for deworming; information provision on breast feeding, immunization, health facility delivery, Birth preparedness and complication Readiness Plan (BPCRP), Post Partum Family Planning (PPFP) and Post Natal Care (PNC) and second visit information were assessed.

**Fig 1:**
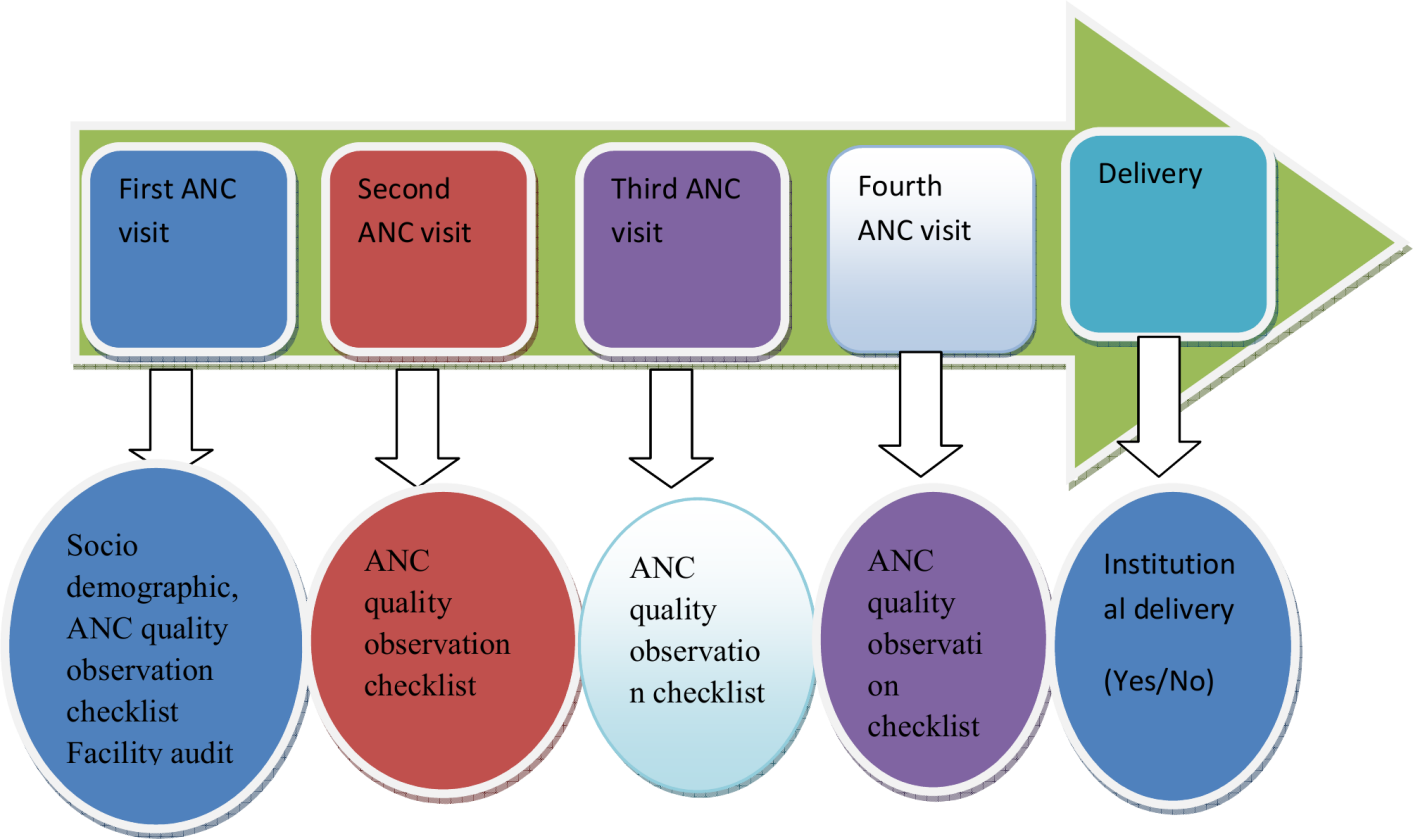
Follow up time for study participants, Bahir Dar, Ethiopia; October 2015 to August 2016.

For fourth visit:history of complaints in current pregnancy; clinical examination and information provision similar to visit three was assessed (Fig 1) Seven female diploma midwives were data collectors that observe about the contents of the ANC services given and the information provided by the ANC provider using the observation check list that was developed based on FANC protocol while the pregnant women received the ANC services. Two BSc female Midwives were recruited as supervisors. Both data collector and supervisors were not working in health facilities under the study. Training was given on the data collection instrument and how to approach and observe the service provision. Pretest of the instrument, close supervision and daily checkup of the filled questionnaire for completeness were also done to maintain the data quality.

Dependent- Institutional delivery (Yes/No)

Independent- Quality of ANC was the main exposure variable.

Definition of quality was adapted based on the frameworks of Donabedian model. Since “structure” is mainly considered as the conduit through which care takes place and “satisfaction” is a consequence of care rather than true components of quality of care [11, 12] the focus of this study was therefore on process attributes of quality. It was measured by the extent to which the pregnant women received the essential ANC services in four visits using 88 items. If the essential ANC service was given it was coded as 1 otherwise 0 and a composite index for the overall ANC service quality was calculated for each woman with a minimum of 0 and maximum of 88 scores. Percentile was computed to categorize the quality of the services a woman received. If she scored ≥ 75^th^ percentile, of essential ANC services, ANC service was considered acceptable quality otherwise not [13].

### Data analysis

Data were coded and entered into EPI data version3.1 and exported to SPSS version 20 for analysis. Descriptive statistics were used to describe the data. Generalized estimating equation analysis (which is an extension of the generalized linear model to allow analysis of correlated observations such as clustered data) with binary response variable using robust estimator and exchangeable working correlation matrix was carried out to control the cluster effect of the data among women who received ANC services within the same facility by the same ANC provider and to identify the predictor variables for institutional delivery. The model fitness was checked by observing the difference of the -2 log likelihood ratio between the null model and the model with independent variables (P value = 0.81). The significance of each independent variable in the equation was also assessed by Wald statistics test at a significance level of P-value < 0.05. In addition, Multico linearity diagnosis was also carried out using variance inflationfactor (VIF), which was <10 for all variables. Based on Hosmer and Leme show applied logistic regression guide a p-value <0.2 was considered to select eligible variables for multivariable regression analyses and p-value <0.05 was considered to identify statistically significant predictor variables for institutional delivery.

## Results

### Background characteristics of ANC attendants

Among 970 enrolled mothers 823 (84.8%) completed the follow up (from their first ANC visit to delivery). The reasons for 15.2% loss to follow up were abortion, self referral to other health facilities run by government, private or NGOs, permanent change in work place, temporary move to their mother’s home for delivery (for nuli para) and long distance to reach the health facilities especially when gestation age increases. There was no statistically significant difference in background characteristics and quality of services received between those who had loss to follow up and those who completed the follow up (quality of the service for those loss to follow up was considered by the services received only in the first visit)

Among women who completed follow up, those with age <19 years (teen age pregnancy) and those with age ≥ 35 years (elderly pregnancy) were 4.9% each. The mean age (±standard deviation) was 25.46±4.37 years. Nearly ninety percent of the women (88.5%) were urban residents and above half of the participants (51.4%) were house wives. About ninety percent, (90.9%) of the study participants were Orthodox Christian and almost all women (98.5%) were currently married. With regard to their educational status, about 17% of them could not read and write while 57.5% of them had attended secondary school and above (Table 1).

**Table 1:** Socio-demographic characteristics of ANC attendants in public health facilities of Bahir Dar city administration (n = 823), October 2015 to August 2016.

Socio-demographic variables		Number	Percent
Age (in years)	15–19	40	4.9
	20–24	314	38.2
	25–29	307	37.3
	30–34	122	14.8
	≥ 35	40	4.9
Religion	Orthodox	748	90.9
	Muslim	73	8.9
	Protestant	2	0.2
Marital status	Married	811	98.5
	Divorced	10	1.2
	Widowed	2	0.3
Educational status	Cannot read and write	139	16.9
	Can read and write	34	4.1
	Grade1-4	61	7.4
	Grade5-8	116	14.1
	Grade9-10	165	20.0
	Grade11-12	152	18.5
	Joined higher institution	156	19.0
Ethnicity	Amhara	788	95.7
	Tigre	25	3.0
	Agew	7	0.9
	Oromo	3	0.4
Occupation	House wife	423	51.4
	Merchant	113	13.7
	Gov’t employee	98	11.9
	Farmer	89	10.8
	Private employee	69	8.4
	NGO employee	5	0.6
	Other*	26	3.2

Other* = daily laborers and waiters

### Obstetric history of ANC attendants

Greater than two out of five of them (42.8%) were nuli para; 26.1% primi para, 30.0% multi para and only 1.1% of them were grand multi para (parity greater or equal to five).

### Adherence to Focused Antenatal Care (FANC) protocol by the health providers

#### Comprehensive history taking

The past obstetric history taking was heterogeneous. Among 471 women with previous history of pregnancy observed, more than 95% of them were asked about their history of hypertension, cesarean section, heavy bleeding and anemia but only 4.2% of women were asked about their history of convulsion (Table 2).

**Table 2:** Comprehensive history taking about past obstetric history among study participants observed at public health facilities of Bahir Dar city administration (n = 471), October 2015 to August 2016.

Past obstetric Hx asked	Number	Percent
Hx. of hypertension	465	98.7
Hx. of heavy bleeding	462	98.1
Hx. of anemia	454	96.4
Hx. of obstructed labour	452	96.0
Hx. of C/S	452	96.0
Hx. of abortion	321	68.2
Hx. of instrumental delivery	151	32.1
Hx. of Multiple pregnancy	86	18.3
Hx. of Still birth	66	14.0
Hx. of convulsion	20	4.2

NB: Hx = history; C/S = Caesarian Section

#### Information provision on danger signs of pregnancy and other ANC services

**Fig 2:**
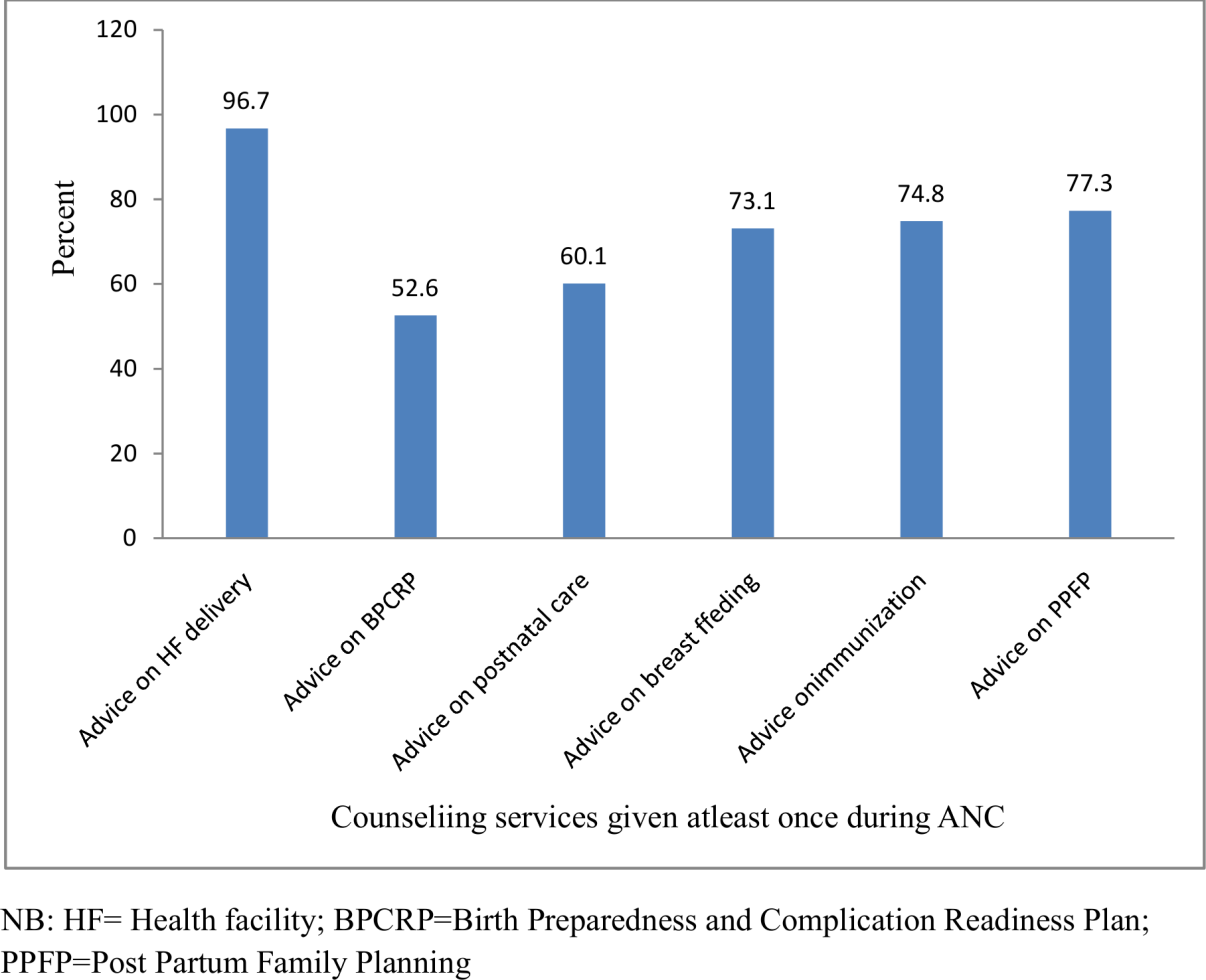
Counselling services provided to observed pregnant women during ANC visits at public health facilities of Bahir -Dar city administration (n = 823), October 2015 to August 2016.

Pregnancy complications and their inadequate management threaten the survival and health of too many mothers and babies. The Safe Motherhood Initiative advocates the provision of advice during antenatal care about potential pregnancy complications, and specifically about how to seek medical care for pregnant women and their families when the danger signs occur. However the information provision and counseling service during antenatal care in the study facilities was not implemented consistently by ANC providers (Table 3 and Fig 2)

**Table 3:** Advice on danger signs of current pregnancy at each ANC visit among study participants observed at public health facilities of Bahir–Dar city administration (n = 823), October 2015 to August 2016.

Danger signs asked	Visit one, n (%)	Visit two, n (%)	Visit three, n (%)	Visit four, n (%)
Vaginal bleeding	610(74.1)	669(81.3)	513(62.5)	554(67.3)
Severe headache	448(54.4)	441(53.6)	772(93.8)	7732(88.9)
Convulsion	27(3.3)	29(3.5)	73(8.9)	83(10.1)
Severe abdominal pain	483(58.7)	345(41.9)	408(49.6)	422(51.3)
Reduced or absent fetal heart beat	-	823(100.0)	823(100.0)	823(100.0)
Facial or hand swelling	224(27.2)	398(48.4)	790(96.0)	706(85.9)
High temperature	487(59.2)	453(55.0)	472(57.4)	434(52.7)

#### Measurements and laboratory tests done during ANC visits

**Fig 3:**
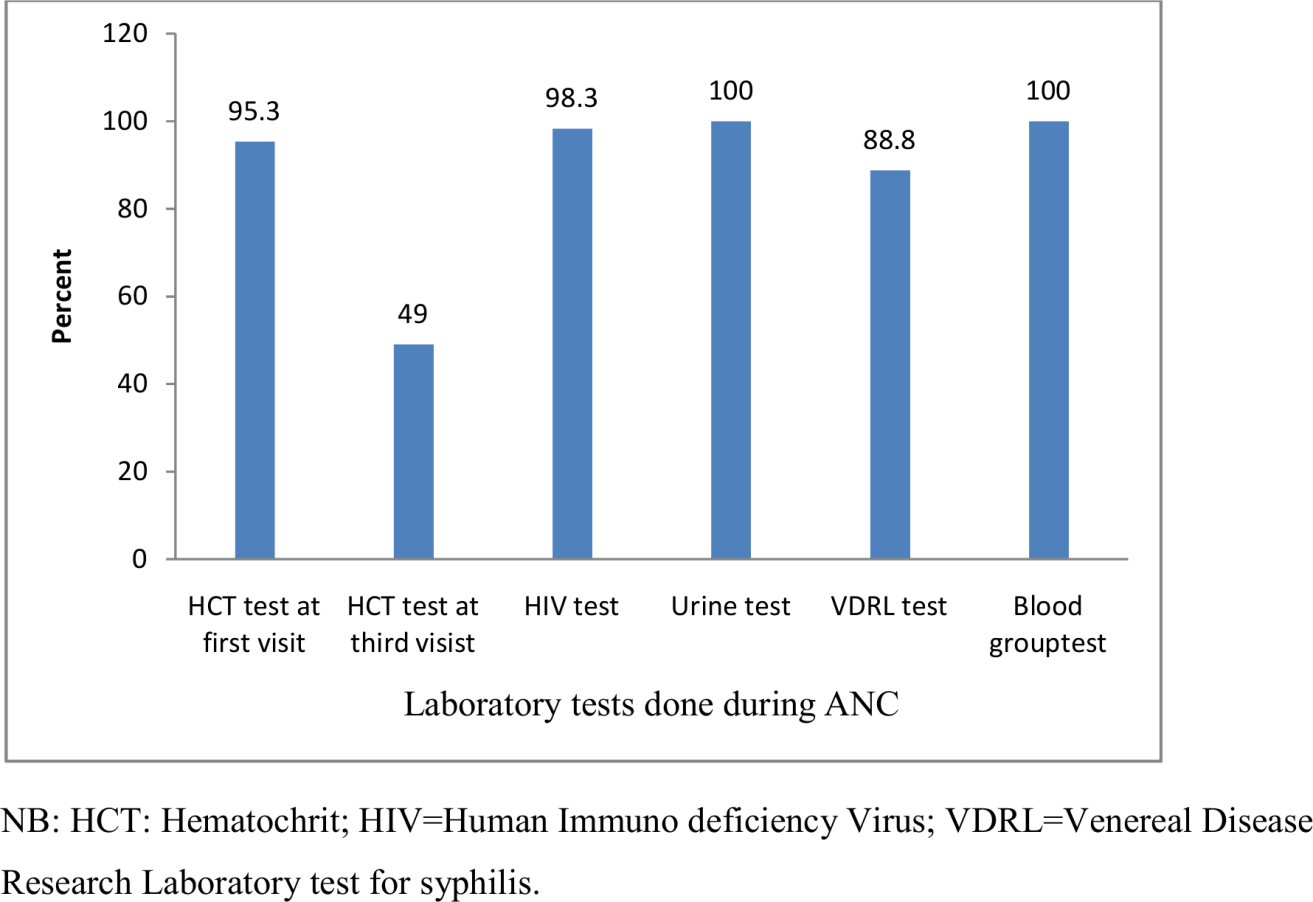
Routine laboratory tests done for pregnant women during ANC visits at public health facilities of Bahir -Dar city administration (n = 823), October 2015 to August 2016.

Weight, blood pressure, fetal heart beat measurements and routine ANC laboratory tests including HIV test were done for ≥ 95.0% of pregnant women. However, HIV test was done in fragmented times (from first visit till delivery) due to inconsistent supply of the test kit. Venereal Disease Research Laboratory (VDRL) test was not done at all for the last two years except only for those pregnant women who were suspected to have syphilis based on history were referred to private health facilities to have the test. More than one in two pregnant mothers (51%) have no hematocrit update at the fourth visit due to the reason that providers repeat the test only if they suspect the women might have anemia (for diagnostic purpose) (Fig 3)

#### Facility audit

Only two public health facilities in had permanently assigned ANC providers while the rest five health facilities assigned providers for ANC, PNC and delivery rooms to work on rotation. The total number of providers who were working in those five facilities by rotation was 12. On the average, each health facility had two providers for ANC and PNC service provision at the same room. Among the 17providers 14(82.4%) were mid wives whereas 3(17.6%) were nurses. Furthermore 8 (47.1%) of the ANC providers were not trained on FANC protocol while 9(52.9%) of them were trained. In all health facilities ANC and PNC services were provided in the same room.

Only one health facility had generator as an alternative power supply when there is no electricity and none of the health facilities had ANC guide line except two health facilities that posted the ANC algorism on the wall inside the ANC room. On the other hand, water in the ANC room and soap were available only in two health facilities.

All health facilities had examination coach, BP apparatus, stethoscope, fetoscope, adult weight scale, dipstick test strips for protein and glucose, electrical centrifuge for hematocrit, Rapid Plasma Reagin (RPR) test kit (except one health facility), blood group test reagents, HIV test kit, HCG test strip, TT, Mebendazole, standardized ANC chart and ANC registration log book.

#### Linkage of antenatal care quality and institutional delivery

The quality of antenatal care was measured by the level of essential antenatal care services given to pregnant women during their 1^st^ to 4^th^ ANC visit with respect to the FANC protocol. Among all pregnant women observed only 227 (27.6%; 95% CI = 24.5%, 30.5%) received acceptable quality of ANC while the rest 596(72.4%; 95%CI = 69.5%, 75.5%) got unacceptable quality of ANC services.

After Hosmer and Lemeshow model adequacy was checked (P-value = 0.81) the binary generalized estimating equation (GEE) logistic regression showed that there was a positive association between antenatal care quality and the use of institutional delivery service; age of the mother, place of residence, occupation, educational status and parity also showed an association with institutional delivery and they were eligible for the multivariable GEE logistic regression analysis.

In multivariable GEE logistic regression, quality of antenatal care, age of the mother, place of residence and educational status of the mother remained to have an association with giving birth at health facility. However, occupation and parity of pregnant women did not show statistically significant association with institutional delivery

The odds of giving birth at health institution among pregnant women who received acceptable ANC quality service was about 3.38 times higher than pregnant women who received un acceptable ANC quality service (AOR = 3.38, 95% CI: 1.67, 6.83).

The odds of giving birth at health facilities among younger pregnant women (<25 years of age) was 3.69 higher than older pregnant women (age ≥ 35 years) (AOR = 3.69, 95% CI: 1.44, 9.49).The odds of giving birth at health facilities among pregnant women living in urban areas was 9.91 times higher than pregnant women who live in rural areas (AOR = 9.91, 95% CI: 2.52, 38.91). Educational status of pregnant women had also positive association with health facility delivery; the odds of giving birth at health facility among pregnant women with educational status of secondary and above was 6.83 times higher compared to those women with no formal education (AOR = 6.83, 95% CI; 3.33,13.97). However, the odds of giving birth at the health facilities among pregnant women who attend primary school and those who had no formal education (AOR = 1.80, 95% CI; 0.97,3.35) did not show statistically significant difference (Table 4).

**Table 4:** Multivariable generalized estimating equation logistic regression to identify determinants of institutional delivery among pregnant women attending ANC at public health facilities of Bahir- Dar city administration (n = 823), October 2015 to August 2016.

Variables		Health Facility Delivery, n (%)	Home Delivery, n (%)	COR(95%CI)	AOR(95% CI)
ANC quality	Acceptable	215 (94.7)	12 (5.3)	5.41(4.34,6.75)	3.38(1.67, 6.83)*
	Not acceptable	508 (85.2)	88 (14.8)	1.00	1.00
Age	15–24 years	319(90.11)	35(9.9)	3.46 (1.59, 7.52)	3.69(1.44, 9.49)*
	25–34 years	370(86.2)	59(13.8)	2.38(1.13, 5.02)	2.14(0.91, 5.01)
	≥ 35 years	29(72.5)	11(27.5)	1.00	1.00
Resident	Urban	680 (93.4)	48 (6.6)	16.41(10.02,26.87)	9.91(2.52,38.91)*
	Rural	43 (45.3)	52(54.7)	1.00	1.00
Occupation	Employee^a^	164 (95.3)	8 (4.7)	25.11(11.02,57.21)	0.77(0.13,4.59)
	House wife	383 (90.5)	40 (9.5)	10.83(6.42,18.27)	0.73(0.17,3.09)
	Others ^b^	134 (96.4)	5 (3.6)	32.83(12.25,87.96)	1.33(0.23,7.72)
	farmer	41(46.1)	48 (53.9)	1.00	1.00
Education	Secondary and above	455 (96.2)	18(3.8)	1.75 (1.28,2.38)	6.83 (3.33,13.97)*
	Primary school	153(86.4)	24(13.6)	14.48(8.24,25.44)	1.80 (0.97,3.35)
	No formal education	110(63.6)	63(36.4)	1.00	1.00
Parity	Nuli para	328 (93.2)	24 (6.8)	4.96 (3.90,6.32)	0.98 (0.54, 1.75)
	Multi para	395 (83.9)	76 (16.1)	1.00	1.00

* Indicates significant difference at *p*< 0.05

^a^ = Government, private and Nongovernmental organization employees

^b =^ Merchants, students, daily laborers and waiters

NB: AOR = Adjusted Odds Ratio, CI = Confidence Interval and COR = Crude Odds Ratio.

## Discussion

In this study only about one in four of the observed pregnant women received acceptable quality of antenatal care service; this is consistent with a study done in rural Mexico that reported 28.4% received acceptable ANC quality [13]. Even if the proportion of women who received quality antenatal care in this study and the study done in Mexico was almost the same, there is 17 years gap in between two studies which indicates that the quality of ANC service in the study area is far behind. This might be due to the reason that the health facilities in the study area might not be well equipped with the necessary inputs and competent professionals for quality ANC service provision. The facility audit also supported the low quality of ANC service in the study area as almost half of ANC providers were not trained on FANC protocol; one health facility did not do syphilis screening test (VDRL/RPR) for the last two years due to lack of the test kit and no health facility had the focused antenatal care guide line.

The quality of ANC service is critical to ensure the intended benefits for both the mother and the baby otherwise the whole process loses credibility. It is one of the interventions that reduce morbidity and mortality risks for the mother and child during pregnancy, delivery, and the postnatal period. Hence achieving the sustainable development goal of reducing global Maternal mortality to <70/100,000 live birth or the target of no country should have a maternal mortality rate > 140/100,000 by 2030 cannot be achieved without quality antenatal care provision [14–16].

Though, the number of women who received quality antenatal care service was low, those who received acceptable quality of antenatal care services with respect to the focused antenatal care protocol had increased odds of giving birth at health facility. This association suggests that a high level of adherence to quality antenatal care by complying with the focused antenatal care protocol could promote skilled institutional delivery, which is directly related to reductions in maternal mortality. This is supported with other study findings [13, 17, 18].

This might be due to the fact that a better quality and packages of ANC services provided to pregnant women may increase their expectations and enthusiasms for future use of the continuum of care like to give birth at health institution and to have postnatal care follow up. Similarly, ANC service quality allows early detection of obstetric complications and gives an opportunity to influence women`s decision to have a skilled attendant during child birth.[19]

In addition, if women received quality ANC service, there is a tendency to be provided with information on the importance of giving birth at health facility and having a birth preparedness and complication readiness plan during emergency conditions like during labor. Especially in Ethiopia where home delivery is the norm (almost three–fourth of the mothers are giving birth at home) [7] planning in advance for delivery event is not part of traditional practice and health facilities are sought as a last resort after serious complications have developed. Hence, if pregnant women are counselled during ANC to have a plan for facility delivery together with arrangement for transport and other amenities like money, blood and companions for emergency conditions; there by highly likely to give birth at health facility.

Furthermore, if women receive quality ANC they will have better knowledge and information on the benefits of accessing maternal health care services thereby increasing their state of preventive health care practice and are more likely to go to the health institutions for delivery before complications occurred.

Other studies also reported women who had three or more visits were more likely to have skilled birth attendant as compared to those who didn’t have follow up[20–22].

This might be due to the fact that; as the frequency of ANC visits increased the packages of ANC services and the information provided to pregnant women by ANC providers might increase their knowledge on the importance of having skilled birth attendant at birth there by increasing the women’s likely hood to give birth at the health facility.

The current study also showed that there were substantial inequalities in the use of health facility delivery service by residence and educational status of the mother. Urban women were more likely to give birth at health facility compared to the rural women. This is consistent with other studies [7, 20, 23].

The possible reasons might be due to urban women tend to have better access to health facilities and can reach to the health facility easily in urgent conditions like during labor as they are accessible to transportation but a rural mother who is in labor and without access to transport will be obliged to walk on foot for a long distance and may further limit their likely hood to give birth at the health institution. Moreover, rural women are more readily influenced by traditional practices that run contrary to modern health care and accept fate about whatever might happen when they delivered at home.

This study also revealed as the educational status of a mother increases beyond secondary school the odds of giving birth at the health institution also increases. This is consistent with other studies [7, 24–26].

This might be explained by educated women are more likely to have a better income and able to afford the costs associated with the health care. compared to uneducated women In addition education may also increase self-confidence and enhance their level of autonomy and freedom to make health-related decisions, including maternal services [27].

This study also revealed that younger pregnant women (<25 years) had higher odds of giving birth at the health facility compared to older pregnant women (≥ 35 years) but when the age of women became 25 years and above it had no significant effect on the odds of giving birth at health institution. This finding is in line with a study done in Andhra Pradesh that reported older pregnant women with age ≥ 30 years were more likely to give birth at home [28]. However this finding is contradictory to a study in India that reported age had no impact on the decision to undergo health facility delivery [29]. The possible reasons for this difference might be due to the reason that older women might have an experience of giving birth before (became multi para) and might have a belief that subsequent pregnancies will result in successful outcome thereby increasing their chance to give birth at home.

However this study reported that parity and occupation of the mother had no statistically significant effect on health facility delivery service use.

As this study was a prospective observational follow up study it avoids recall and social desirability biases unlike that of retrospective follow up studies In addition employing a generalized estimating equation analysis controls the clustering effect among women who received ANC services within the same facility by the same ANC provider. To increase the generalizability of the study findings, adequate sample size was calculated and the participants were selected using aprobability sampling method which was systematic sampling method; thus, selection bias was unlikely to happen or its effect would be minimal.All these could be mentioned as strength of the current study.

However, ANC providers might work with their maximum effort while the data collectors observed their service provision (Hawthorn effect) even if its effect might slowly decrease when the providers adapt their presence as the data collection period was long (one year). To minimize the bias data collectors did not tell the ANC providers about what specific ANC services they were observing rather than telling them in general way. Though midwifery professional data collectors and standardized observation checklist adapted from FANC protocol was used to minimize observation bias, its effect might not be zero due to a difference in their competency.

Lack of qualitative study component to explore the cultural factors affecting the use of health facility delivery as delivery is highly culturally held event could also be the limitation of this study.

### Conclusion and recommendations

The quality of antenatal care service in public health facilities of the study area was compromised/low and adherence to FANC protocol had a great role in promoting the use of health facility delivery. However, the provision of essential ANC services was heterogeneous.

Provision of acceptable quality of ANC service, being urban dweller, being younger age and having higher educational status of pregnant women were the predictors that increase the use of institutional delivery. Hence the local authorities at each level of health sector or the nongovernmental organizations working to improve maternal health need to provide trainings for ANC providers on FANC protocol focusing on the importance of information provision on BPCRP, danger signs of pregnancy, comprehensive history taking on past & current obstetric history. In addition, ANC providers should be committed to comply with the focused antenatal care protocol so that no mother might not be missed without informing the danger signs, BPCRP and other recommended ANC services giving emphasis for rural and lower educational status women and they also need to request their necessary inputs before they become out of stock. Moreover the Federal Ministry of Education should work towards universal access for education beyond primary education level by giving emphasis for females.

## Supporting information

S1 Dataset Data set for a study on the link between quality of ANC service and institutional delivery. (SAV)

## Abbreviations

ANC: Antenatal Care
AOR: Adjusted Odds Ratio
BPCRP: Birth Preparedness and Complication Readiness Plan
CI: Confidence Interval
COR: Crude Odds Ratio
FANC: Focused Antenatal Care
GEE: Generalized Estimating Equation
SPSS: Statistical Packages for Social Sciences
VDRL: Venereal Disease Research Laboratory
